# Autoimmune thyroiditis associated with neuromyelitis optica (NMO)

**DOI:** 10.3205/000226

**Published:** 2015-11-18

**Authors:** Sreenivasa Rao Sudulagunta, Mahesh Babu Sodalagunta, Hadi Khorram, Mona Sepehrar, Jayadevappa Gonivada, Zahra Noroozpour, Nagendra Prasad

**Affiliations:** 1 Columbia Asia Hospital, Bangalore, India; 2 K.S. Hegde Medical College, Mangalore, India; 3 Dr.B.R. Ambedkar Medical College, Otolaryngology Department, Bangalore, India; 4 Baptist Hospital, Bangalore, India; 5 Dr.B.R. Ambedkar Medical College, Bangalore, India

**Keywords:** neuromyelitis optica (NMO), optic neuritis, transverse myelitis, IgG, autoimmune thyroiditis

## Abstract

Neuromyelitis optica (NMO or Devic’s syndrome) is a rare relapsing demyelinating disease of the central nervous system (CNS) that mainly affects the spinal cord and optic nerves and shares many clinical and radiological features with multiple sclerosis. The association of NMO with other autoimmune diseases was reported, but very few reports described association with autoimmune thyroid disease. Early differentiation between NMO and multiple sclerosis is very important as the natural course and treatment regimens differ significantly. We report a case of a 50-year-old woman who was admitted initially with vomiting, hiccups and paraesthesias but was not diagnosed with NMO and presented with a severe progression of the disease. The patient was also diagnosed to have autoimmune thyroiditis with lymphocytic infiltration of the thyroid which progressed from hyperthyroidism to hypothyroidism. NMO diagnosis was established with seropositivity for NMO-IgG and MRI showing longitudinally extensive spinal cord lesions (3 or more spinal segments). In spite of treatment, the response was poor due to lack of early diagnosis and aggressive immunosuppressant therapy.

## Zusammenfassung

Neuromyelitis optica (NMO) oder Devic-Syndrom ist eine seltene, rezidivierende, entmyelinisierende Erkrankung des Zentralnervensystems, die das Rückenmark und die Sehnerven angreift und viele klinische und radiologische Symptome mit der multiplen Sklerose teilt. Eine Assoziation der NMO mit anderen Autoimmunerkrankungen wurde bereits veröffentlicht, aber nur wenige Berichte beschreiben die Verbindung zur autoimmunen Thyreoiditis. Die frühe Unterscheidung zwischen NMO und multipler Sklerose ist wichtig, weil die Verläufe und die Therapieverfahren deutlich verschieden sind. Wir berichten über eine 50 Jahre alte Patientin, die anfangs mit Erbrechen, Schluckauf und Parästhesien eingewiesen wurde. Die NMO war nicht erkannt worden, und die Erkrankung zeigte eine schwere Ausprägung. Bei der Patientin wurde auch eine autoimmune Thyreoiditis mit Lymphocyteninfiltraten festgestellt. Die Schilddrüsenfunktion wandelte sich von einer Hyperthyreose zu einer Hypothyreose. Die NMO-Diagnose wurde gesichert mit seropositiven NMO-IgG und MRI-Untersuchungen, mit denen extensive longitudinale Rückenmarksläsionen (in 3 und mehr Segmenten) nachgewiesen wurden. Der Behandlungserfolg war gering, bedingt durch das Fehlen einer frühen Diagnose und einer aggressiven immunsuppressiven Therapie.

## Introduction

Neuromyelitis optica (NMO or Devic’s syndrome) is a rare relapsing autoimmune disease of the central nervous system that preferentially affects the spinal cord and optic nerves and shares many radiological and clinical features with multiple sclerosis (MS) [1], [2], [3]. NMO is characterized by longitudinally extensive transverse myelitis (LETM, myelitis affecting 3 vertebral segments in length or more), and optic neuritis which can be unilateral or bilateral. The factors that differentiate it from MS include the following characteristics:

1. involvement of the brain is rare, specifically early in the disease,
2. the severity of attacks is more robust than MS, and
3. the pathophysiology differs – MS is a T cell mediated disease, while NMO is mediated by anti-aquaporin-4 antibodies.

NMO is now considered a disease entity rather than a subtype of MS after discovery of a novel, pathogenic autoantibody (termed NMO-IgG or AQP4-Ab) in 2004 [4], [5]. 

NMO can affect patients as young as 3 years and as old as 90 years, but constitutes less than 1% of demyelinating diseases [6], [7]. Clinical, MRI, and spinal fluid features from several case series are illustrated in Table 1 (Tab. 1). The current approximate estimates of prevalence in Japan has been 14 per 1,000,000, and in North West England 4 per 1,000,000 [8], [9] with a female-to-male ratio of 3:1. The mean age of onset is approximately 40 years, but cases also have been reported in childhood [2]. Devic’s disease is more common in Asians and Africans compared to Caucasians. The Mayo Clinic proposed a revised set of criteria for diagnosis of Devic’s disease in 2006 (Table 2 (Tab. 2)) [10].

Hashimoto’s thyroiditis or chronic lymphocytic thyroiditis is an autoimmune disease characterized by cell- and antibody-mediated immune processes against the thyroid gland, causing primary hypothyroidism. The occurrence of this condition is estimated to be 1 to 1.5 in 1,000 people [11]. Hashimoto’s disease is often associated with family members having thyroid or other autoimmune diseases and sometimes with having other autoimmune diseases of the patients themselves [12]. 

Diagnosis of Hashimoto’s thyroiditis requires observations of lymphocytic infiltration of the thyroid [13] and autoantibodies against thyroid peroxidase, thyroglobulin, and uncommonly thyroid hormone stimulating receptors [14]. Pathogenesis may be caused by a molecular mimicry mechanism, abnormal antigen-specific induction of T cells due to abnormal HLA-related SPC (sphingosylphosphorylcholine) genes, mutation of T cells to form abnormal clones, or an immune defect causing reduced induction of T suppressor cells by specific antigens. 

## Case report

A 50-year-old woman was admitted on January 2014 with complaints of vomiting, hiccups and generalized fatigue for 11 days, paraesthesias of bilateral upper limbs and lower limbs for 5 days. As the patient was a type 2 diabetic for 7 years, peripheral neuropathy was diagnosed in local hospital and treatment was given. She became asymptomatic after 15 days. Her investigations (complete blood count, differential count, renal function tests, liver function tests, B12 levels, folic acid levels, hemoglobin) were normal during that time. However, HBA1C was 9.8, fasting blood sugar was 300 mg/dl and postprandial blood sugar was 416 mg/dl for which dosage of insulin was increased. The patient was not evaluated with MRI or CSF analysis and was managed for diabetic neuropathy during the first episode.

The thyroid profile was abnormal, suggestive of hyperthyroidism for which she refused to take medication (Table 3 (Tab. 3)). No history of smoking, illegal drug use or alcohol consumption was noted. No history of fever, cough and shortness of breath was noted. No history of hypertension, heart disease and other autoimmune disorders was obtained. In August 2014, the patient was admitted with weakness of bilateral lower limbs and upper limbs along with a band-like sensation around the chest at the level of T4 vertebra. The patient also complained of visual disturbances in the form of difficulty in seeing distant objects in bilateral eyes right > left. The CSF analysis done in the government neurology center was found to be normal during the episode.

A MRI scan of brain and spine showed increased T2 signal and expansion of the cord in some areas of the spinal cord at the levels of C2–C5, and C7 to T12. The patient was given methylprednisolone 1 gram intravenous for a period of 5 days and oral steroids for 4 weeks. Thyroid function tests were reported to be normal (Table 3 (Tab. 3)). The patient showed marginal improvement in vision and reduction of sensory abnormalities by about 50% over a period of 6 weeks. In April 2015, the patient was admitted with cough associated with mucopurulent expectoration, shortness of breath, paraesthesias and diminished vision. The patient showed minimal improvement in sensory symptoms or vision. In May 2015, the patient was admitted with progression of bilateral lower limb weakness, bilateral upper limb weakness (distal > proximal) and a band-like sensation around the chest, syncope, vomiting and hiccups. Clinical examination revealed normal higher mental functions with diminished vision to finger counting in the right eye. Other cranial nerves were normal.

Hypertonia was noted in all limbs. The power was 2/5 bilaterally in the proximal upper limbs and distal muscles. The lower limb power was 1/5 on admission, but improved to 2/5 in 15 days. Upper limb reflexes were 2+ and knee and ankle reflexes were 2+. The Babinski reflex was present bilaterally. Loss of joint position and vibration sense till the lower boarder of the sternum was noted. THe abdominal reflex was absent. Antibodies to HSV1, HSV2, CMV, EBV, HBV, VZV, HCV and HIV in serum and cerebrospinal fluid, as well as sarcoidosis and tumor markers in serum revealed no abnormality. Polymerase chain reaction in CSF for HSV1 and HSV2 was negative. Cerebrospinal fluid analysis demonstrated no oligoclonal bands. 

**Figure 1:**
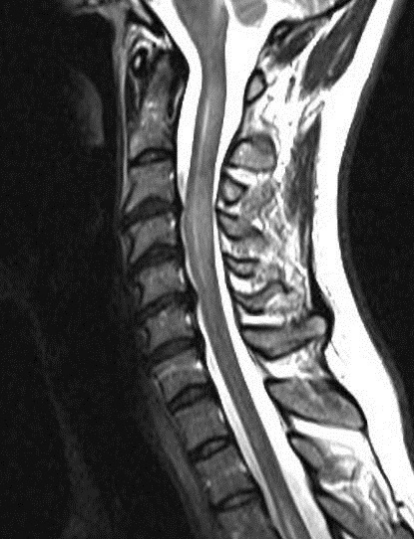
MRI spine showing hyperintensity in cervical cord C5 to C7 level (1)

**Figure 2:**
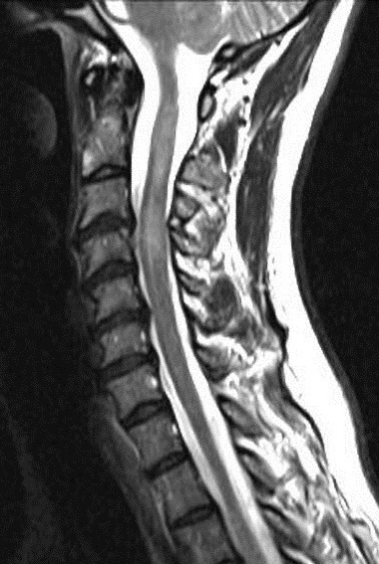
MRI spine showing hyperintensity in cervical cord C5 to C7 level (2)

**Figure 3:**
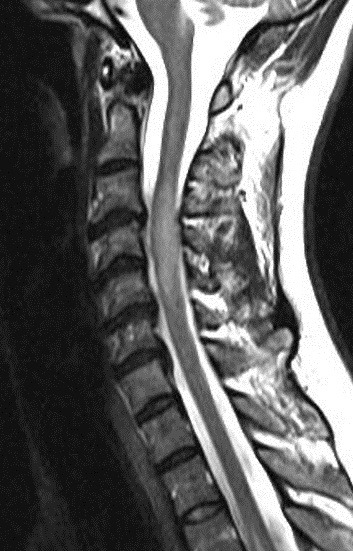
MRI spine showing hyperintensity in cervical cord C5 to C7 level (3)

**Figure 4:**
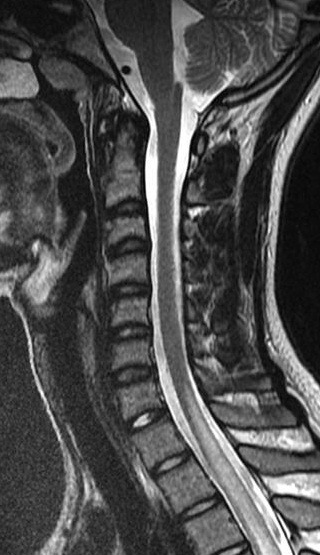
MRI spine showing hyperintensity in the thoracic cord till T12 level (1)

**Figure 5:**
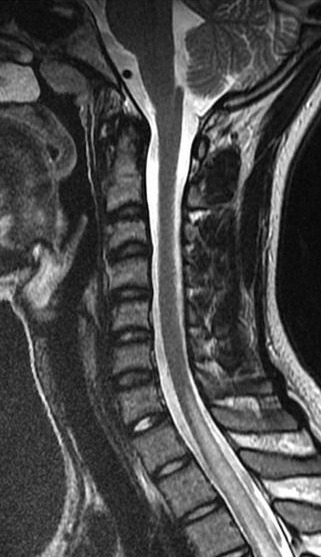
MRI spine showing hyperintensity in the thoracic cord till T12 level (2)

**Figure 6:**
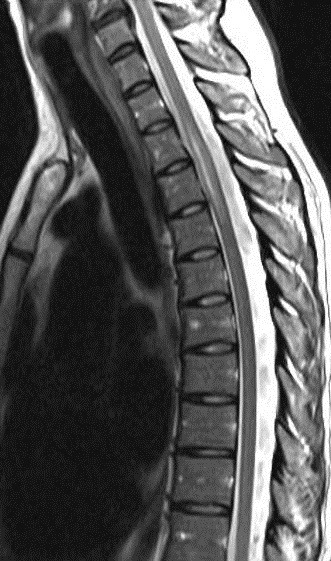
MRI spine showing hyperintensity in the thoracic cord till T12 level (3)

**Figure 7:**
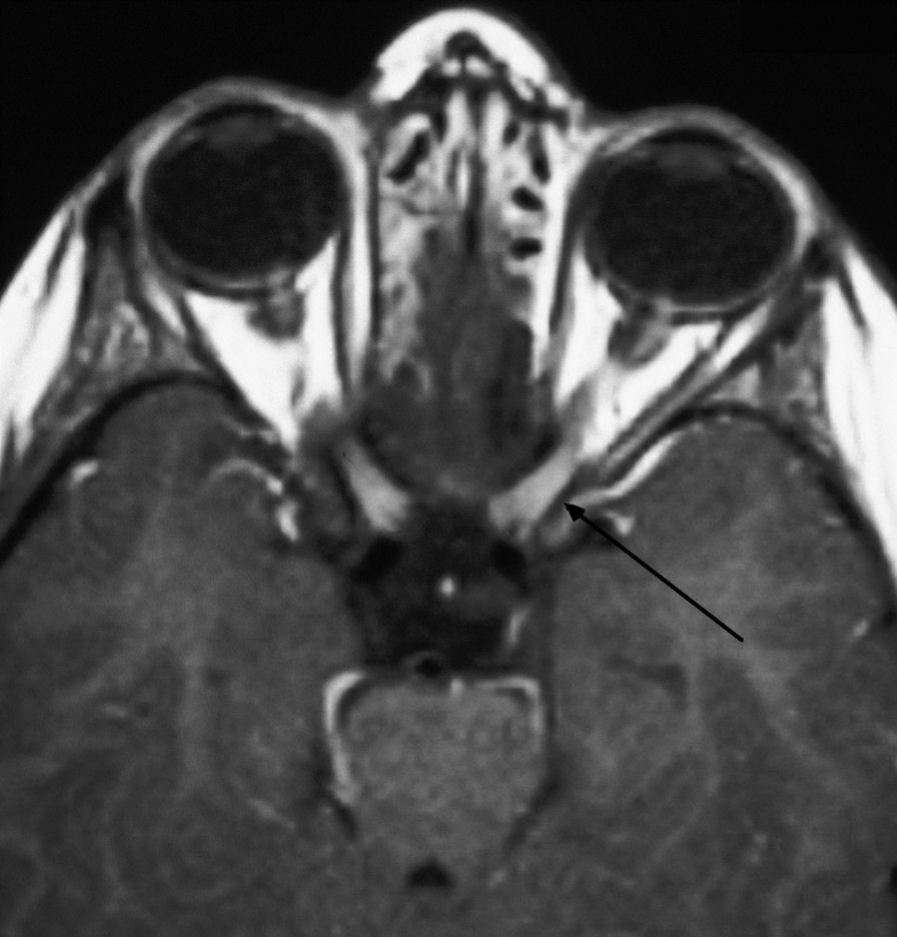
MRI brain showing hyperintensity in the right optic nerve head

**Figure 8:**
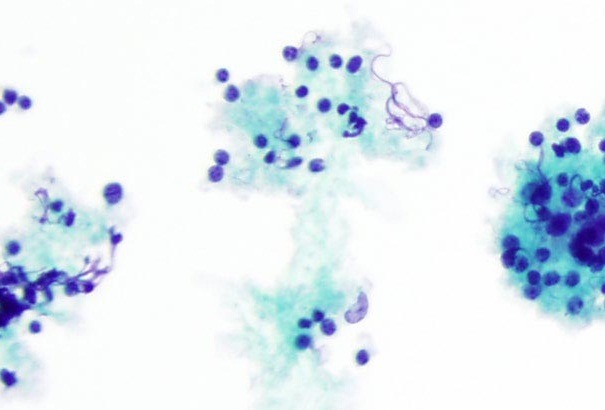
Fine needle aspiration of thyroid showed lymphocytic infiltration of thyroid gland

**Figure 9:**
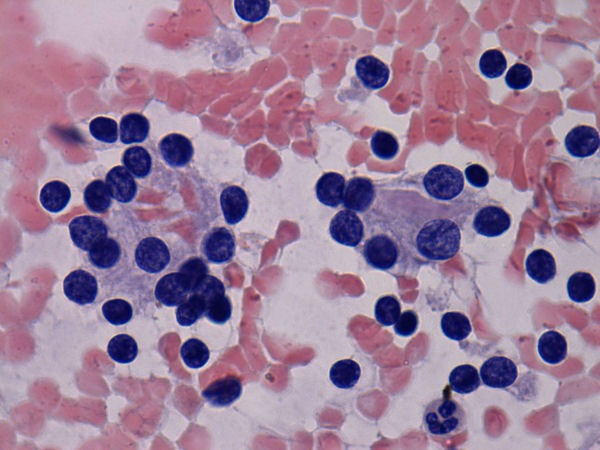
Fine needle aspiration of thyroid showed lymphocytic infiltration of thyroid gland

Immunological tests for ANA titer were 1:320, tests for anti-ENA, anti-dsDNA, anti-cardiolipin, anti-β2GPI, lupus cells, antibodies to GAD65, IA-2, insulin and cryoglobulins were negative. MRI brain and whole spine revealed 1) hyperintensity in cervical cord C5 to C7 level (Figure 1 (Fig. 1), Figure 2 (Fig. 2), Figure 3 (Fig. 3)); 2) hyperintensity in the thoracic cord till T12 level (Figure 4 (Fig. 4), Figure 5 (Fig. 5), Figure 6 (Fig. 6)); 3) hyperintensity in the right optic nerve head (Figure 7 (Fig. 7)). The clinical features and investigations (NMO IgG was positive) fit into the criteria of neuromyelitis optica. The thyroid profile was suggestive of hypothyroidism (Table 3 (Tab. 3)). Laboratory studies confirmed the presence of antithyroid antibodies (Table 4 (Tab. 4)). Fine needle aspiration of the thyroid showed lymphocytic infiltration of the thyroid gland (Figure 8 (Fig. 8), Figure 9 (Fig. 9)). The patient was treated with prednisolone 1 g/day for 5 days and azathioprine (2.5–3 mg/kg/daily). The patient showed improvement in motor symptoms and sensory symptoms approximately by 60% after a period of 2 months of treatment. Patient was started on thyroxine 50 μg and later dosage was increased to 100 μg.

## Discussion

Devic’s disease is a severe idiopathic immune-mediated inflammatory demyelinating disease that preferentially involves the spinal cord and optic nerves. More than 90% of patients with NMO have repeated relapses rather than monophasic disease. Clinical events can occur simultaneously or can be separated by long intervals of months to years. Several differences exist in the characteristics and outcomes of patients with the monophasic and relapsing forms (Table 5 (Tab. 5)) [2]. Systemic autoimmune diseases are associated commonly with NMO compared to MS. Oligoclonal bands are seen in 85–90% of MS cases but only 20–30% of NMO cases. 

The NMO-IgG autoantibody is highly specific (91%; 85–99%) and sensitive (73%; 58–76%), and has less frequent occurrence in MS [15]. Its target antigen is the AQP4 water-pump channel; an integral protein of astrocytic plasma membranes and is highly concentrated in the astrocyte foot processes. The distribution of AQP4-rich areas in the central nervous system, especially in the central spinal cord, hypothalamus, periventricular area and periaqueductal areas is highly indicative of NMO lesions [16].

Spinal cord histopathology in NMO found loss of AQP4 in acute inflammatory lesions surrounding immunoglobulin and complement-deposited hyalinized small vessels which suggests humorally mediated microangiopathy leading to spinal cord lesions in NMO. High anti-AQP4 antibody titers are associated with complete blindness and correlate positively with the length of spinal cord lesions on MRI. Anti-AQP4 antibody titers decrease after high-dose methylprednisolone, and follow-up shows low titers in relapse-free periods under immunosuppressive treatment.

Autoimmune diseases encompass a wide spectrum of diseases from organ specific (Hashimoto’s thyroiditis) to various systemic diseases including systemic lupus erythematosus (SLE) characterized by inflammation and production of autoantibodies detected against multiple autoantigens. Etiology is poorly understood but, genetic, immunological, hormonal and environmental factors have a role. A patient suffering from one autoimmune disease has a 25% chance of acquiring another autoimmune disease which is found in our patient.

Studies reported a strong association of NMO with systemic autoimmune diseases, including SLE or Sjögren syndrome (SS), or non-organ-specific autoantibodies (e.g. antinuclear antibody, extractable nuclear antigen) [2], [17]. Even though neurological complications of autoimmune diseases like SLE and SS have been reported for a long time [18], [19], [20], [21], the relationship between them and CNS inflammatory demyelinating disorders such as multiple sclerosis, transverse myelitis and NMO have been poorly understood.

Few Western studies have described patients with manifestations of NMO (transverse myelitis and optic neuritis) and systemic autoimmune diseases [22], [23], [24]. Association of NMO and autoimmune thyroiditis was reported very rarely [25]. The role of immunosuppressive treatment in neuromyelitis optica is well established. The role of this treatment in associated autoimmune diseases is not clearly established.

Long-term immunosuppressive treatment is required to prevent relapses in patients (Table 6 (Tab. 6)). The current drugs used are corticosteroids, intravenous immuglobulin (IVIG), azathioprine, rituximab, mitoxantrone, mycophenolate mofetil and interferon beta etc. [26] with several trials supporting use of immunosuppressive drugs (Table 7 (Tab. 7)). There are no prospective randomized clinical trials offering class I evidence to direct therapy for relapse prevention [27]. Treatment decisions are largely guided by case series and expert opinions.

Through our case report we put forward the following observations. Awareness of autoimmune diseases in neuromyelitis optica should be increased. Early diagnosis and aggressive immunosuppressive treatment is important in the management of NMO. IgG NMO testing is very useful in diagnosis, even if clinical and para-clinical autoimmune indices are available.

## Notes

### Competing interests

The authors declare that they have no competing interests.

## Figures and Tables

**Table 1:**
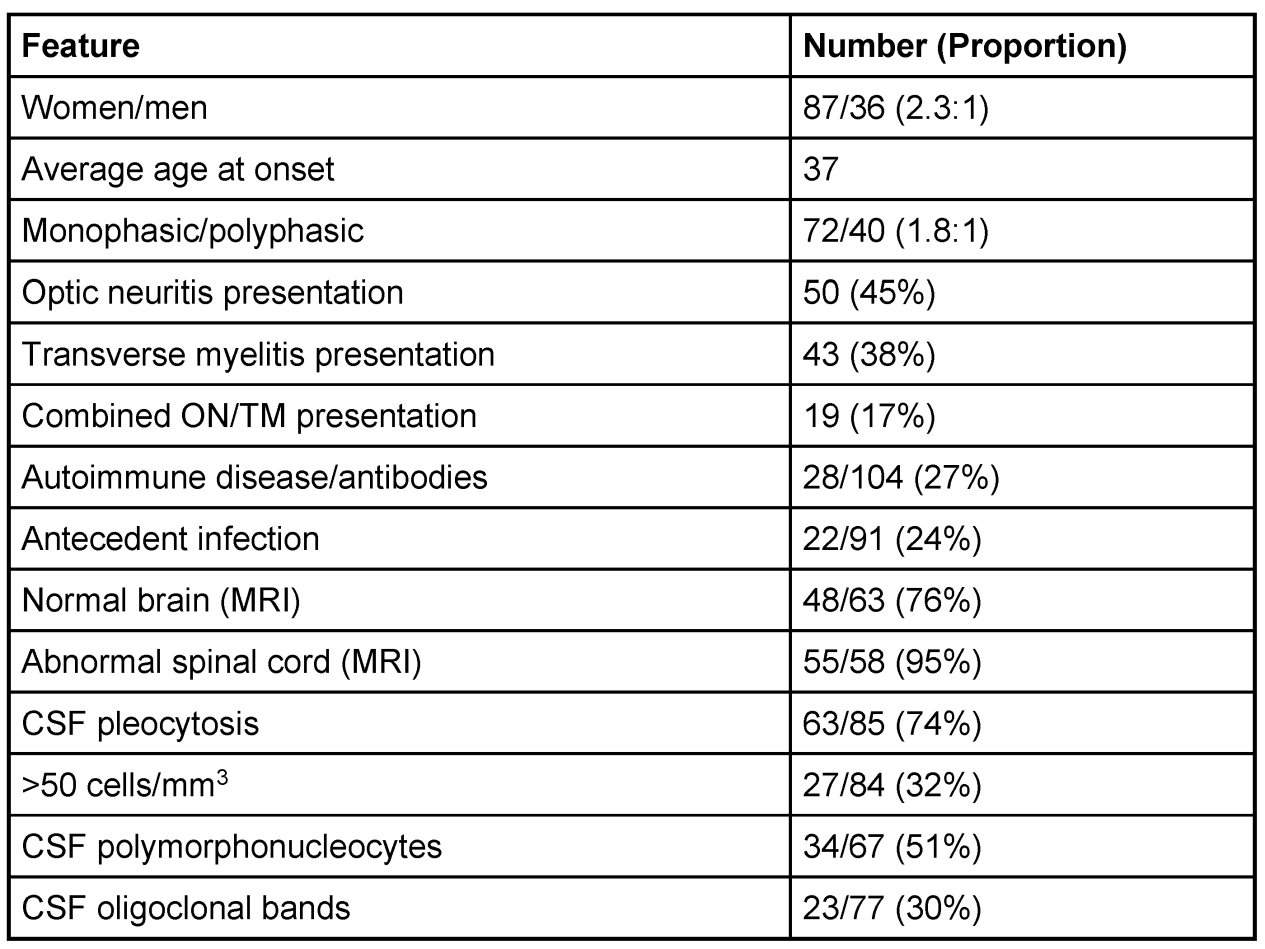
Clinical, MRI, and spinal fluid features from several case series

**Table 2:**
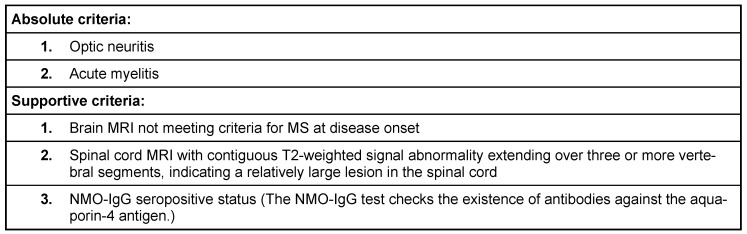
Mayo clinic criteria for NMO

**Table 3:**
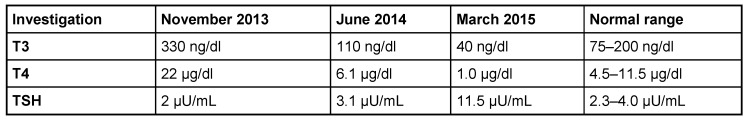
Thyroid profile

**Table 4:**
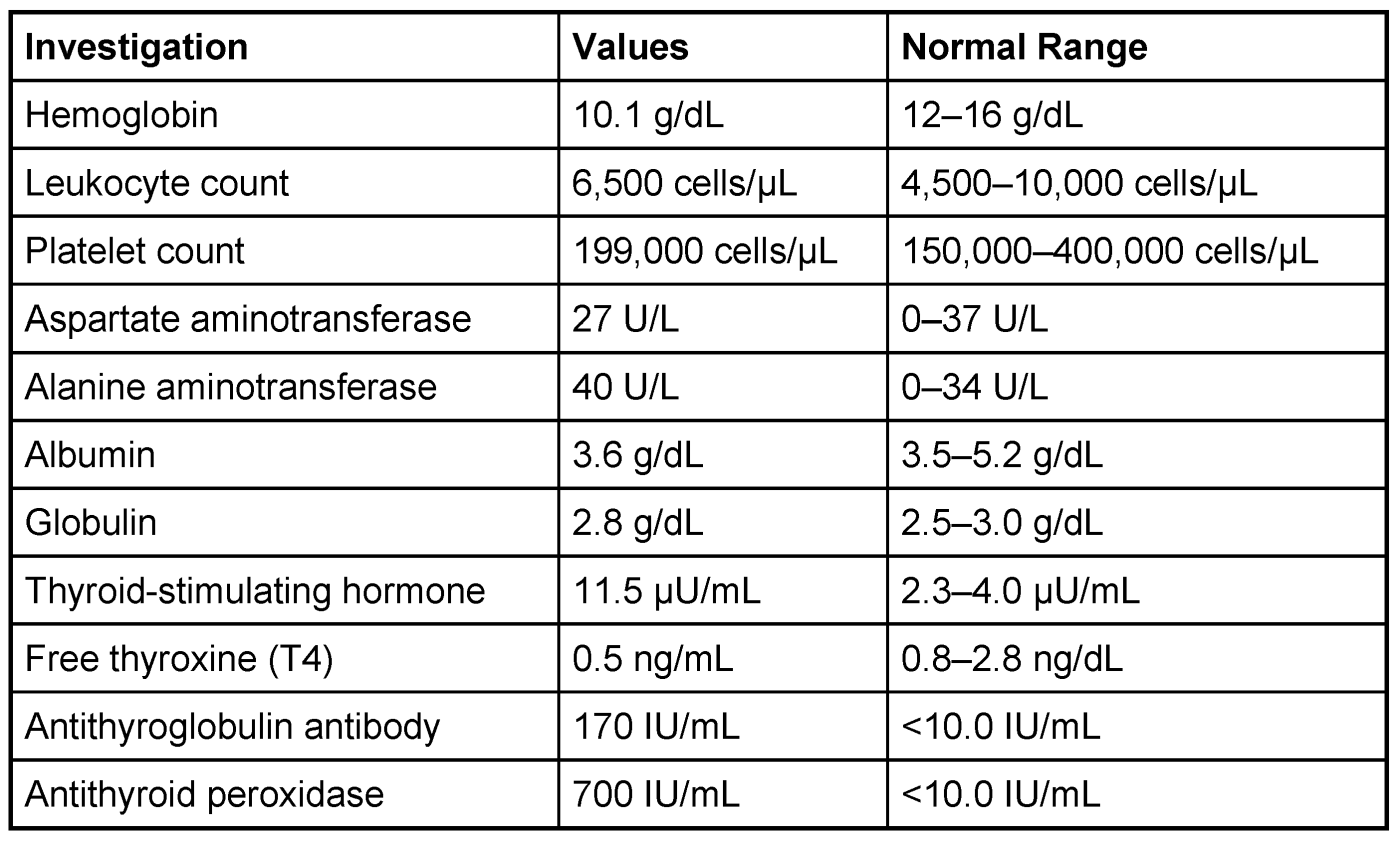
Laboratory studies

**Table 5:**
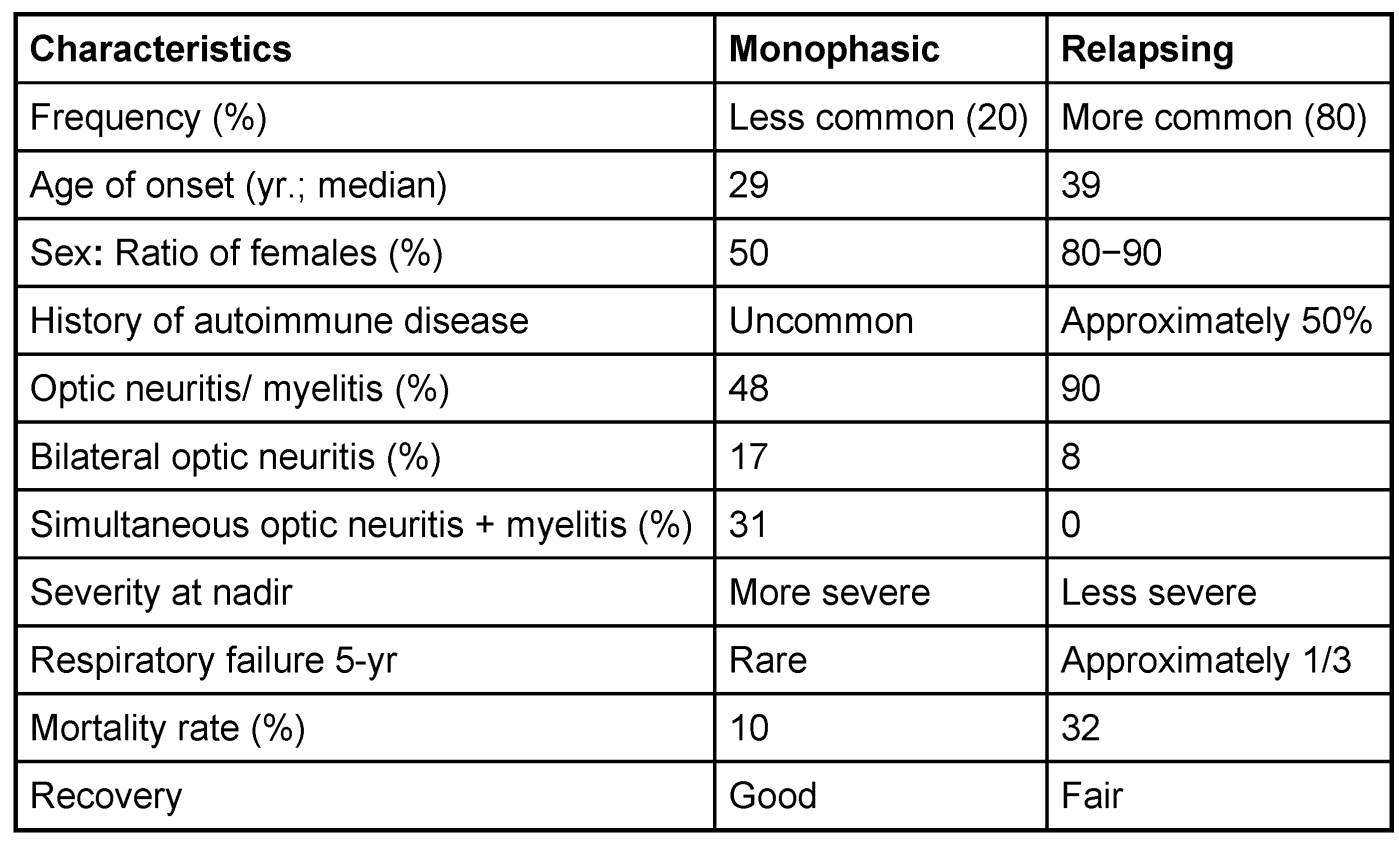
Characteristics of monophasic and relapsing neuromyelitis optica

**Table 6:**
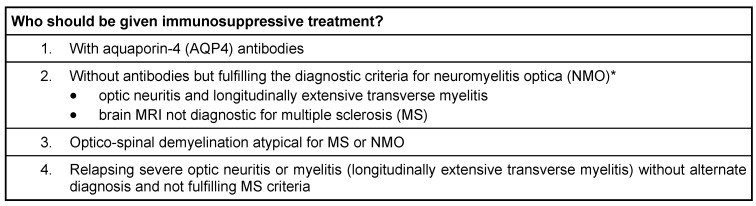
Immunosuppressive treatment

**Table 7:**
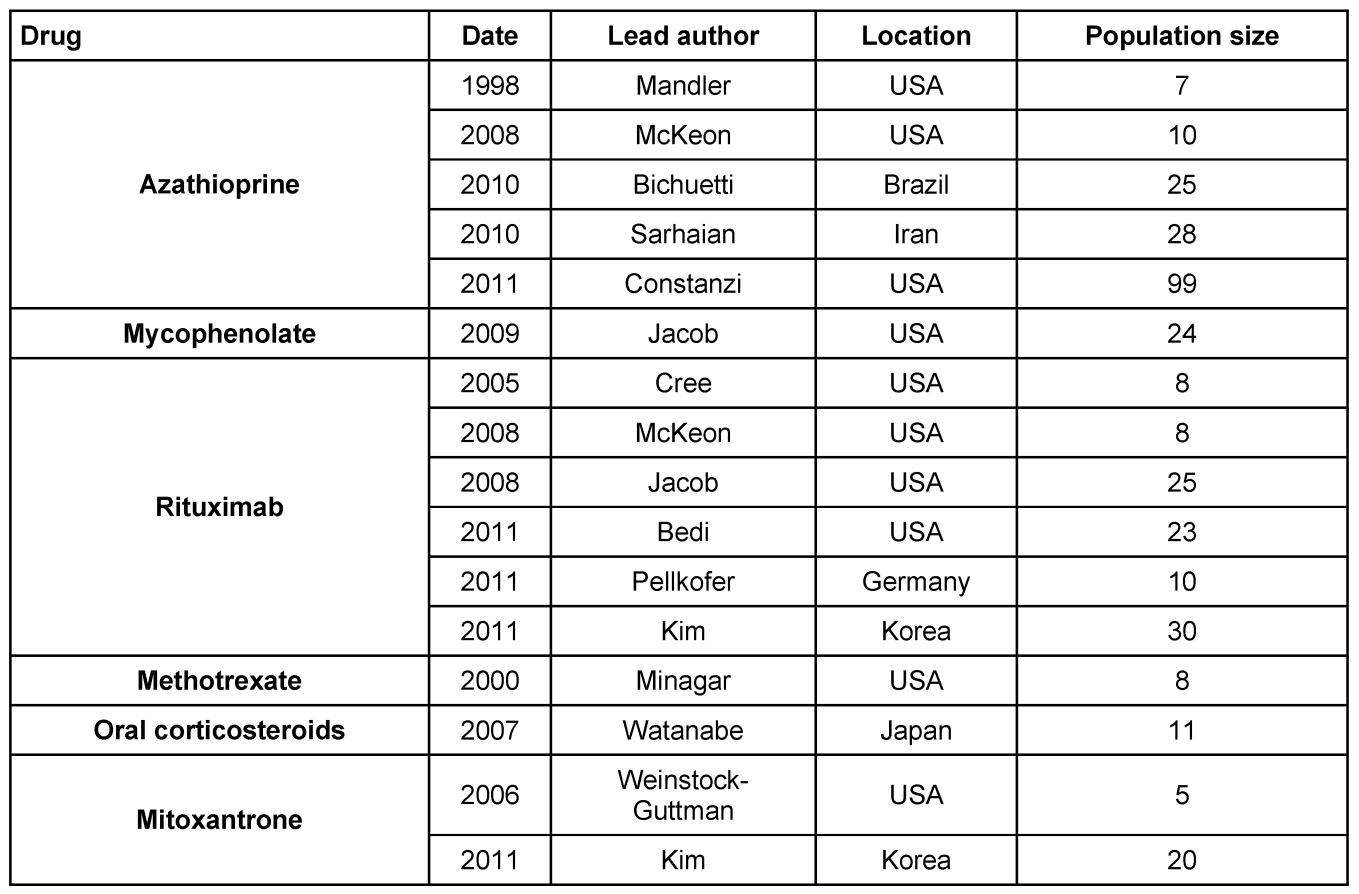
Trials supporting immunosuppressive therapy in NMO
